# Supplementary material for: Influenza A Virus Infection Reactivates Human Endogenous Retroviruses Associated with Modulation of Antiviral Immunity
Source: Viruses. 2022 Jul 21;14(7):1591. doi: 10.3390/v14071591 (PMC9320126; doi:10.3390/v14071591)
Supplement: Supplementary file 1 [file viruses-14-01591-s001.zip › MotifAnalysis.zip!/MotifOutput_SC35M/homerResults.html]

MotifOutput// - Homer de novo Motif Results


# Homer *de novo* Motif Results (MotifOutput//)

Known Motif Enrichment Results  
Gene Ontology Enrichment Results  
If Homer is having trouble matching a motif to a known motif, try copy/pasting the matrix file into
STAMP  
More information on motif finding results: HOMER
| Description of Results
| Tips
  
Total target sequences = 41  
Total background sequences = 47386  
\* - possible false positive  

|  |  |  |  |  |  |  |  |  |
| --- | --- | --- | --- | --- | --- | --- | --- | --- |
| Rank | Motif | P-value | log P-pvalue | % of Targets | % of Background | STD(Bg STD) | Best Match/Details | Motif File |
| 1 | C G T A A C G T C T G A A G T C C G T A A G T C A G T C G T C A T A C G A C G T | 1e-20 | -4.701e+01 | 31.71% | 0.44% | 57.4bp (65.0bp) | SNAI3/MA1559.1/Jaspar(0.705) More Information \| Similar Motifs Found | motif file (matrix) |
| 2 | A T C G C G T A A C T G C G T A G T A C G T C A A G T C C G A T A C G T A C G T | 1e-17 | -4.112e+01 | 31.71% | 0.70% | 61.6bp (75.0bp) | PB0117.1\_Eomes\_2/Jaspar(0.612) More Information \| Similar Motifs Found | motif file (matrix) |
| 3 | A C G T A T G C A C G T A C T G A C T G A C G T A T C G C A T G A C T G A C T G G T C A A G T C | 1e-17 | -3.952e+01 | 26.83% | 0.38% | 62.8bp (84.3bp) | RUNX2(Runt)/PCa-RUNX2-ChIP-Seq(GSE33889)/Homer(0.668) More Information \| Similar Motifs Found | motif file (matrix) |
| 4 | C G T A A G T C A G T C C T G A C G T A A C G T A G T C C T A G C T A G A G T C | 1e-16 | -3.786e+01 | 24.39% | 0.29% | 40.7bp (80.8bp) | NFYA/MA0060.3/Jaspar(0.847) More Information \| Similar Motifs Found | motif file (matrix) |
| 5 | A G T C A G C T T C A G A C G T C T A G A C G T G A T C A C G T C G T A A C T G A G T C C G A T | 1e-16 | -3.771e+01 | 24.39% | 0.29% | 49.0bp (86.9bp) | Smad4/MA1153.1/Jaspar(0.685) More Information \| Similar Motifs Found | motif file (matrix) |
| 6 | C T G A A C T G A C T G C G A T A C G T A C G T A C T G A C G T C T G A C G T A | 1e-15 | -3.642e+01 | 21.95% | 0.20% | 47.5bp (87.6bp) | SRY/MA0084.1/Jaspar(0.644) More Information \| Similar Motifs Found | motif file (matrix) |
| 7 | C G T A A C G T A G T C G T A C A C G T A C T G A G C T C G T A A T G C G T C A G T A C A G T C | 1e-15 | -3.539e+01 | 19.51% | 0.12% | 65.2bp (71.4bp) | ZBTB32/MA1580.1/Jaspar(0.686) More Information \| Similar Motifs Found | motif file (matrix) |
| 8 | C G T A A C G T A G T C A C G T A G T C C G T A A C G T A C G T A C T G A G T C A G T C A C T G | 1e-13 | -3.151e+01 | 9.76% | 0.00% | 58.0bp (13.3bp) | Hoxa10(Homeobox)/ChickenMSG-Hoxa10.Flag-ChIP-Seq(GSE86088)/Homer(0.684) More Information \| Similar Motifs Found | motif file (matrix) |
| 9 | C G T A A G T C A C G T A C G T A C T G A G C T A C T G A T C G | 1e-13 | -3.041e+01 | 41.46% | 3.87% | 56.7bp (71.1bp) | RUNX2(Runt)/PCa-RUNX2-ChIP-Seq(GSE33889)/Homer(0.732) More Information \| Similar Motifs Found | motif file (matrix) |
| 10 | A G T C C G A T C G T A A C T G A T G C A C G T G T C A C G T A A C T G A C T G | 1e-13 | -3.015e+01 | 21.95% | 0.40% | 47.8bp (74.2bp) | GATA4/MA0482.2/Jaspar(0.732) More Information \| Similar Motifs Found | motif file (matrix) |
| 11 | A C T G C G A T A C G T G T A C A G T C G T C A A C G T A G T C A C G T C G T A A G T C A C G T | 1e-12 | -2.876e+01 | 12.20% | 0.02% | 54.2bp (63.7bp) | ZKSCAN1/MA1585.1/Jaspar(0.602) More Information \| Similar Motifs Found | motif file (matrix) |
| 12 | A G T C A G T C A G T C A G T C C G T A A C T G A G T C A G T C A G T C A C T G C G T A A G T C | 1e-12 | -2.874e+01 | 9.76% | 0.01% | 55.3bp (24.3bp) | KLF4/MA0039.4/Jaspar(0.676) More Information \| Similar Motifs Found | motif file (matrix) |
| 13 \* | A C T G A C T G A C T G G T A C G T C A A G T C C T A G A G T C A C G T A G T C A G T C C G T A | 1e-11 | -2.743e+01 | 17.07% | 0.18% | 15.7bp (57.6bp) | PAX5/MA0014.3/Jaspar(0.673) More Information \| Similar Motifs Found | motif file (matrix) |
| 14 \* | C G T A C G T A C G T A C G T A C G T A A G T C G T A C A C T G A G T C A G T C A G T C A C G T | 1e-11 | -2.712e+01 | 9.76% | 0.01% | 45.2bp (39.4bp) | MYB(HTH)/ERMYB-Myb-ChIPSeq(GSE22095)/Homer(0.653) More Information \| Similar Motifs Found | motif file (matrix) |
| 15 \* | A C T G C T G A C G T A C T G A A C G T C T G A A G T C A G T C A C G T A C T G C T A G A G C T | 1e-11 | -2.708e+01 | 12.20% | 0.03% | 51.3bp (81.7bp) | Prdm15/MA1616.1/Jaspar(0.665) More Information \| Similar Motifs Found | motif file (matrix) |
| 16 \* | A C G T A G T C A G T C A C G T A G T C A C G T A C G T A C T G A C G T G T C A A C G T A G T C | 1e-11 | -2.642e+01 | 12.20% | 0.03% | 21.4bp (69.5bp) | IKZF1/MA1508.1/Jaspar(0.614) More Information \| Similar Motifs Found | motif file (matrix) |
| 17 \* | G T A C A T G C A G T C A T G C A T G C G T C A G A T C A T C G A C G T A C G T G T C A G T A C | 1e-11 | -2.575e+01 | 17.07% | 0.23% | 38.6bp (75.8bp) | PB0201.1\_Zfp281\_2/Jaspar(0.677) More Information \| Similar Motifs Found | motif file (matrix) |
| 18 \* | C T G A A G T C C T G A A G T C A G C T G T A C A G C T A C T G | 1e-11 | -2.565e+01 | 31.71% | 2.38% | 55.5bp (66.7bp) | POL013.1\_MED-1/Jaspar(0.697) More Information \| Similar Motifs Found | motif file (matrix) |
| 19 \* | A G T C A C G T G T C A G C T A A G C T A G T C G T A C G T A C A G T C A G T C A C G T A T G C | 1e-10 | -2.530e+01 | 12.20% | 0.04% | 46.0bp (52.3bp) | Crx/MA0467.1/Jaspar(0.720) More Information \| Similar Motifs Found | motif file (matrix) |
| 20 \* | A G T C A G T C A G T C A G C T A C G T C G T A C G A T A G C T C G T A C T A G A C T G A C T G | 1e-10 | -2.502e+01 | 14.63% | 0.12% | 53.6bp (81.8bp) | CArG(MADS)/PUER-Srf-ChIP-Seq(Sullivan\_et\_al.)/Homer(0.702) More Information \| Similar Motifs Found | motif file (matrix) |
| 21 \* | A G T C A G T C A C G T A C G T A G T C A C G T A C G T A G T C A G T C A G T C C G T A C G T A | 1e-10 | -2.373e+01 | 9.76% | 0.02% | 58.0bp (33.5bp) | PB0124.1\_Gabpa\_2/Jaspar(0.707) More Information \| Similar Motifs Found | motif file (matrix) |
| 22 \* | C G T A A G T C A G T C A G T C A C G T A C G T C G A T C G T A G T A C A C T G A C T G A C T G | 1e-9 | -2.231e+01 | 9.76% | 0.02% | 44.7bp (83.4bp) | MYBL1/MA0776.1/Jaspar(0.583) More Information \| Similar Motifs Found | motif file (matrix) |
| 23 \* | T A G C G T A C G T A C T C A G C G T A G A T C C G A T G A T C G A T C G T A C | 1e-9 | -2.185e+01 | 12.20% | 0.09% | 52.6bp (69.4bp) | PB0203.1\_Zfp691\_2/Jaspar(0.603) More Information \| Similar Motifs Found | motif file (matrix) |
| 24 \* | A C T G A C G T A C T G C G T A A C G T A C T G A G T C A C G T A C T G A C G T A C T G A G T C | 1e-9 | -2.093e+01 | 7.32% | 0.01% | 3.3bp (0.0bp) | PB0207.1\_Zic3\_2/Jaspar(0.691) More Information \| Similar Motifs Found | motif file (matrix) |
| 25 \* | C G T A C G T A A C T G C G T A A C T G A C T G G T C A C T G A A C T G A C T G A G T C G T A C | 1e-8 | -2.019e+01 | 9.76% | 0.04% | 3.0bp (51.9bp) | NKX2-3/MA0672.1/Jaspar(0.634) More Information \| Similar Motifs Found | motif file (matrix) |
| 26 \* | A G T C A C G T A C G T C G T A A C T G A G T C C T G A A C T G C G T A A G T C | 1e-8 | -1.882e+01 | 9.76% | 0.05% | 59.4bp (81.9bp) | NFIC/MA0161.2/Jaspar(0.650) More Information \| Similar Motifs Found | motif file (matrix) |
| 27 \* | C G T A A C G T A G T C A G T C A C T G A C T G C G T A A C T G A C T G A G T C | 1e-7 | -1.818e+01 | 7.32% | 0.01% | 5.2bp (49.0bp) | PB0077.1\_Spdef\_1/Jaspar(0.779) More Information \| Similar Motifs Found | motif file (matrix) |
| 28 \* | A C G T C T A G A G T C A T C G A C G T A G T C C T A G A C T G A C G T C G T A | 1e-7 | -1.793e+01 | 9.76% | 0.06% | 16.1bp (64.9bp) | MafA(bZIP)/Islet-MafA-ChIP-Seq(GSE30298)/Homer(0.621) More Information \| Similar Motifs Found | motif file (matrix) |
| 29 \* | C G T A C G T A C T G A C T A G G A T C G T A C A G C T C T A G C G A T G C A T | 1e-6 | -1.503e+01 | 12.20% | 0.34% | 49.0bp (70.8bp) | GRHL2/MA1105.2/Jaspar(0.733) More Information \| Similar Motifs Found | motif file (matrix) |
| 30 \* | G T A C T C A G A C T G C A G T G T C A C G A T G C T A C G T A | 1e-5 | -1.245e+01 | 7.32% | 0.07% | 2.9bp (68.3bp) | PB0059.1\_Six6\_1/Jaspar(0.627) More Information \| Similar Motifs Found | motif file (matrix) |
| 31 \* | A C T G C T A G A G T C A C G T A G T C A G T C A G T C A C G T A C T G A C G T | 1e-5 | -1.188e+01 | 12.20% | 0.65% | 51.6bp (71.0bp) | Ets1-distal(ETS)/CD4+-PolII-ChIP-Seq(Barski\_et\_al.)/Homer(0.698) More Information \| Similar Motifs Found | motif file (matrix) |
| 32 \* | A C T G C G T A A C G T C G T A A G T C A G T C A C G T A G T C | 1e-5 | -1.154e+01 | 12.20% | 0.69% | 52.3bp (77.0bp) | PB0126.1\_Gata5\_2/Jaspar(0.804) More Information \| Similar Motifs Found | motif file (matrix) |
| 33 \* | C G T A C G T A A C G T A C T G A C G T C G T A A G T C A C G T A C G T A C G T A C T G A C G T | 1e-4 | -9.539e+00 | 4.88% | 0.03% | 39.5bp (40.1bp) | SOX10/MA0442.2/Jaspar(0.720) More Information \| Similar Motifs Found | motif file (matrix) |
| 34 \* | A T C G A G T C A C G T A C G T A C G T C G A T C G T A C G T A C G A T A C T G | 1e-3 | -8.666e+00 | 7.32% | 0.26% | 31.1bp (66.2bp) | TATA-Box(TBP)/Promoter/Homer(0.746) More Information \| Similar Motifs Found | motif file (matrix) |
